# The Role of Synthetic Biology in Atmospheric Greenhouse Gas Reduction: Prospects and Challenges

**DOI:** 10.34133/2020/1016207

**Published:** 2020-07-28

**Authors:** Charles DeLisi, Aristides Patrinos, Michael MacCracken, Dan Drell, George Annas, Adam Arkin, George Church, Robert Cook-Deegan, Henry Jacoby, Mary Lidstrom, Jerry Melillo, Ron Milo, Keith Paustian, John Reilly, Richard J. Roberts, Daniel Segrè, Susan Solomon, Dominic Woolf, Stan D. Wullschleger, Xiaohan Yang

**Affiliations:** ^1^Department of Biomedical Engineering and Program in Bioinformatics, College of Engineering, Boston University, Boston MA 02215, USA; ^2^The NOVIM Group, Kohn Hall, UC Santa Barbara, CA 93106USA; ^3^Climate Institute, Washington, DCUSA; ^4^Department of Energy, Washington, DC, USA; ^5^Center for Health Law, Ethics & Human Rights at the Boston University School of Public Health, School of Medicine, Boston University, USA; ^6^Department of Bioengineering, University of California, Berkeley CA, USA; ^7^Department of Genetics, Harvard Medical School, Cambridge MA, USA; ^8^School for the Future of Innovation in Society, Arizona State University, Barrett & O’Connor Washington Center, 1800 I Street, NW, Washington, DC 20006, USA; ^9^Sloan School of Management, MIT, Cambridge MA, USA; ^10^Department of Chemical Engineering, University of Washington, Seattle Washington, USA; ^11^The Ecosystems Center of the Marine Biological Laboratory in Woods Hole, MAUSA; ^12^Department of Plant and Environmental Sciences, Weizmann Institute of Science, Rehovot, Israel; ^13^Department of Soil and Crop Sciences, Colorado State University, Fort Collins CO 80523, USA; ^14^MIT Joint Program on the Science and Policy of Global Change, MIT, Cambridge MA, USA; ^15^New England Biolabs, Beverly MA, USA; ^16^Department of Biology and Program in Bioinformatics, Boston University, Boston MA 02215, USA; ^17^Department of Earth, Atmospheric and Planetary Sciences, MIT, Cambridge MA, USA; ^18^Soil and Crop Sciences Section, School of Integrated Plant Sciences, Cornell University, Ithaca NY, USA; ^19^Environmental Sciences Division, Oak Ridge National Laboratory, Oak Ridge TN, USA; ^20^Biosciences Division, Oak Ridge National Laboratory, Oak Ridge, TN, USA

## Abstract

The long atmospheric residence time of CO_2_ creates an urgent need to add atmospheric carbon drawdown to CO_2_ regulatory strategies. Synthetic and systems biology (SSB), which enables manipulation of cellular phenotypes, offers a powerful approach to amplifying and adding new possibilities to current land management practices aimed at reducing atmospheric carbon. The participants (in attendance: Christina Agapakis, George Annas, Adam Arkin, George Church, Robert Cook-Deegan, Charles DeLisi, Dan Drell, Sheldon Glashow, Steve Hamburg, Henry Jacoby, Henry Kelly, Mark Kon, Todd Kuiken, Mary Lidstrom, Mike MacCracken, June Medford, Jerry Melillo, Ron Milo, Pilar Ossorio, Ari Patrinos, Keith Paustian, Kristala Jones Prather, Kent Redford, David Resnik, John Reilly, Richard J. Roberts, Daniel Segre, Susan Solomon, Elizabeth Strychalski, Chris Voigt, Dominic Woolf, Stan Wullschleger, and Xiaohan Yang) identified a range of possibilities by which SSB might help reduce greenhouse gas concentrations and which might also contribute to environmental sustainability and adaptation. These include, among other possibilities, engineering plants to convert CO_2_ produced by respiration into a stable carbonate, designing plants with an increased root-to-shoot ratio, and creating plants with the ability to self-fertilize. A number of serious ecological and societal challenges must, however, be confronted and resolved before any such application can be fully assessed, realized, and deployed.

## 1. Introduction

For nearly three decades after the 1992 Earth Summit in Rio, nations have tried to frame a global regime to control greenhouse gas emissions and to assist with adaptation, yet over this period, emissions have continued to increase. Now, under the 2015 Paris Agreement, all nations have pledged reductions in emissions—though the United States has since withdrawn its commitment—with the goal of limiting global warming below 2°C above preindustrial levels and to seek to hold the change to 1.5°C. Carbon budget studies show that meeting these objectives will require reducing net greenhouse gas emissions (to achieve net zero emissions, CO_2_ must be removed from the atmosphere at nearly the same rate that it enters) virtually to zero from all sectors—including agriculture, land use, and construction, as well as energy—within a few decades, a daunting challenge [1].

Equally important, the temperature objectives of the Paris Accord are not likely to meet the general objective of the UN Framework Convention on Climate Change, which is to stabilize “greenhouse gas concentrations in the atmosphere at a level that would prevent dangerous anthropogenic interference with the climate system.” In particular, we are witnessing major disturbances in important ecosystems, including forests in northwestern North America, the fire-prone southwest US, and the 2020 fires in Australia. Coral ecosystems are being lost. Food production in several regions is being threatened by increasingly frequent cycles of severe precipitation and drought, and sustainable economic development is being disrupted by extreme weather events that were once rare [2, 3].

These environmental changes assume greater significance when one recognizes that even if CO_2_ emissions were to stop immediately, the long-term temperature consequences would remain substantial since the time scale for temperature reduction by natural processes is on the order of a thousand years [4]. CO_2_ emission will, of course, not stop immediately; the extent to which it continues, and therefore the rate at which the postindustrial global average temperature increases, depends on the growth in the world’s future energy use and the types of energy utilized [5] and the management of agricultural land, all of which are unknown.

Given this situation, a number of new technologies have been proposed to remove CO_2_ from the atmosphere [6, 7], including biotic and abiotic methods [8]. These are generally divided into approaches that would enhance natural removal processes and industrial processes that would scrub CO_2_ from the atmosphere and store it in geological formations. The former includes increased uptake of carbon by terrestrial or marine systems, reforestation, and increased storage in soils. latter includes deployment of large structures forThe latter includes deployment of large structures for filtering CO_2_ from flowing air using various chemical sorbents followed by mineral carbonation and storage and use of enhanced mineral weathering. Rather than storage, there is a huge opportunity to supply the captured carbon-to-carbon negative technologies. Significant investment has been made in direct air capture companies (e.g., Climeworks, Carbon Engineering, and Global Thermostat) on this promise, and SSB offers a huge opportunity here. As one example, the use of CO_2_-utilizing bacteria that would directly feed off this technology is discussed below.

Bioenergy with carbon capture and storage (“storage” is sometimes referred to as “sequestration”—both terms mean the same: keeping the CO_2_ from coming back into the atmosphere for many, many centuries), which is prominently discussed in recent accords, would remove atmospheric CO_2_ by growing perennial grasses or trees and then combusting this biomass to generate electrical energy for general use while capturing and sequestering the CO_2_ that is produced [9]. Implementation, however, faces a number of very difficult challenges, including cost, the limited availability of arable land, and the needed water resources and fertilizer necessary for growing biomass.

Although numerous carbon dioxide removal (CDR) strategies have been studied and analyzed in some detail [10], the community has been relatively silent on assessing the potential of one of the most revolutionary technologies of the current century: SSB—the ability to design and program biological systems to carry out prespecified functions. The potential for SSB to modify plants to convert CO_2_ to a stable nonrespirable form rather than returning it to the atmosphere offers a potentially important opportunity to moderate climate [11]. Given the increasingly disruptive impacts of climate change, starting a discussion of the potential benefits and risks of SSB seemed highly desirable. Moreover, if the history of genomics [12] is any indication, sizeable economic returns on any investment, both directly and through cobenefits, might be realized [13].

To begin exploring possibilities, a group of researchers with expertise spanning SSB, atmospheric and terrestrial science, policy, and ethics, met in Boston December 3-4, 2019, to discuss some of the potential applications of SSB and its impacts on sustainability and atmospheric carbon drawdown and to address the technical and social challenges of large-scale implementation. Several interrelated themes emerged, including the following.

(i) There is a potential role that SSB might play in diminishing climate change and its impacts by
  (a) contributing to regional and global sustainability
  (b) drawing down atmospheric carbon or reducing emissions and
  (c) avoiding the release of CO_2_ (or methane) into the atmosphere [14, 15]
(ii) The wide range in time scales that would be needed for proof-of-principle, scaling, and cost reductions depends on the application
(iii) The economic cost-benefit of SSB, which could weigh heavily in favor of benefit as a consequence of substantial cobenefits, including less expensive, more effective crop growth; the ability to use degraded lands that are currently not sufficiently arable for the growth of crops and other useful plant products; the potential for discovery of plant-based pharmaceuticals or biomaterials; and, more generally, a boost in the growth of agrogenomic start-ups
(iv) An emerging roadmap for future research, which includes an open and global assessment of serious ethical, social, legal, environmental, and scientific issues that must be resolved before SSB can be introduced as a climate control measure

## 2. Opinion

We summarize below some of the ways in which the rapidly emerging ability to engineer phenotypes can be developed to address one of the major challenges confronting the planet, viz., climate change—including the associated challenges of sustainability and human health.

### 2.1. Plants Could Potentially Be Engineered to Reduce Atmospheric Carbon Dioxide

SSB can modulate at least some paths of the fast carbon cycle, which moves tens of billions of tons of carbon through the biosphere annually. Roughly 120 gigatons of carbon (GtC) per year are cycled between the atmosphere and terrestrial life and another 90 GtC between the atmosphere and the ocean’s mixed layer. Genetic modification of relevant plant traits (e.g., biomass yield, root system architecture, root depth, lignin content, suberin content, photosynthetic, and water- and nitrogen-use efficiency) to achieve even a small perturbation in the 120 GtC respired to the atmosphere each year can have a pronounced impact on its carbon content. Introducing such changes, however, requires a better understanding of the components and dynamics of ecosystems than we currently have.

### 2.2. Genes That Control the Distribution of Biomass Could Potentially Be Identified, Opening the Way for Genetically Modifying the Root-to-Shoot Ratio and Root Architecture of Plants

Research programs aimed at reducing greenhouse (GHG) emissions by developing efficient and cost-effective plant-based fuels have been in place for decades yet have had relatively limited impact on overall GHG emissions. A more recent pursuit, which on the face of it is different, entails using plants to sequester atmospheric carbon in soil at higher rates than has been possible up until now. The availability of the poplar genome sequence [16] has in principle enabled a connection between the two. In particular, the past two decades have seen progress in identifying genes that control biomass distribution between roots and shoots [17] and in understanding plant/microbe interactions that foster CO_2_ sequestration [18]. This opens the possibility of designing and/or breeding plants that have above-ground properties desirable for developing advanced fuels or improving the sustainable development of agriculture and below-ground properties desirable for carbon sequestration [19]. At the same time, it could help restore carbon-depleted soil.

### 2.3. Plants Could Potentially Be Engineered to Increase Agricultural Productivity and Create a Robust Environment

Plant synthetic biology, although still in its infancy, is proceeding apace. A recent example is the approval of Golden Rice [20–22] by the Philippine Department of Agriculture—an important step in combating malnutrition, as the world’s population continues to grow and climatic disruption continues. In fact, new applications of precision agriculture, which would improve crop yields, increase land sustainability and help the world adapt to a changing planet may well be the best initial and least invasive focus of SSB. Biotechnology can help ameliorate the impacts of climate-induced increases in precipitation extremes in a number of ways; for example, progress has been made in engineering rice that is resistant to flooding and plants with increased resistance to drought.

Recent progress in SSB also promises the possibility of increased agricultural productivity. More than 80% of the Earth’s plant species—including rice, wheat, and soybeans—are subject to photorespiration, a process that reduces yields by more than 50%. However, a number of common plants have evolved photosynthetic adaptations that minimize photorespiration and save water. Examples are corn, which utilizes a series of biochemical reactions known as the C_4_ pathway, and pineapple, which utilizes the crassulacean acid metabolism (CAM) pathway. SSB opens the possibility of moving the C_4_, CAM, or other carbon concentration pathways into various types of plants [23], which would help increase agricultural productivity [24, 25]. As C_3_-to-CAM evolution mainly involves convergent diel gene expression changes, engineering of CAM into C_3_ crop plants can focus on rewiring the diel expression pattern of CAM-related genes that already exist in C_3_ plants, without transferring genes from CAM plants into C_3_ plants [26]. This accelerated C_3_-to-CAM evolution approach based on SSB could reduce the public concern of genetically modified organisms discussed below. In addition, recent work shows that dissipative energy processes in crops (e.g., NPQ, nonphotochemical quenching) that produce 13% reduction in crop carbon assimilation may be targets for substantial enhancement [27].

### 2.4. Plants Could Potentially Be Engineered to Self-Fertilize

Legumes and some trees harbor bacteria in root nodules that convert atmospheric nitrogen gas into plant-accessible nitrogen that is required for growth. This is in contrast to cereals such as oat and wheat, which provide most of the world’s calories, but often require exogenous nitrogen to maximize productivity [28].

Adding N fixation capabilities to nonleguminous crop plants has the potential to reduce denitrification rates and the associated N_2_O production, a powerful long-lived GHG, as well as ecological disruption caused by run-off of water-soluble nitrates and concomitant increases of hypoxia, lack of oxygen, in receiving waters. Also, it is possible to engineer bacteria associated with crops as fertilizers, which is already practiced commercially (e.g., Pivot Bio), though at an early stage of development. It would be interesting to compare these two strategies to see if direct N fixation in crops is advantageous. Production of nitrogen fertilizer which might be reduced through adding N fixation to the rhizospheres of the major agronomic crops would reduce the need for energy-intensive production of N fertilizer using the Haber Bosch process, which accounts for some 2-3% of total global GHG emissions. Although nitrogen fixation in root nodules does not eliminate N_2_O production, emission factors are significantly lower than for mineral-N fertilizer [29].

### 2.5. Algae, Ferns, and Other Photosynthetic Organisms Could Potentially Facilitate Atmospheric CO_2_ Drawdown

Trees, which store the majority of terrestrial plant carbon, are genetically complicated and slow growing. A decade or more of research would likely be required just for proof of principle of a tree-based carbon drawdown strategy, e.g., the conversion of some organic carbon to a stable form such as calcium carbonate, rather than respiring it back into the atmosphere. Alternatively, a strategy using crops such as beans, peas, and other N-fixing legumes or, as indicated above, even cereals that are engineered to fix atmospheric nitrogen might, with adequate funding, be proven sooner.

Another type of photosynthetic organism, the Azolla fern, is believed to have played a major role in the CO_2_ reduction that started some 60 million years ago [30]. Azolla grows extremely rapidly, making it of particular interest when time constraints are severe, as they now are. Recently, two fern genomes, including a species of Azolla, have been sequenced [31]—in part through direct public support—perhaps paving the way for understanding the genetic basis of rapid proliferation and for engineering relevant pathways into other organisms.

A better understanding of climate-ecosystem dynamics is a prerequisite to potentially harnessing any drawdown strategy. An example of the kind of study that is needed is underway in the Arctic [32]. At least some portions of the Arctic appear to be much richer in photosynthetic capacity than past field experiments indicated [33] and might potentially contribute to achieving negative emissions. Serious containment and governance problems must be solved, however, before any technology can be implemented, as discussed below. Small-scale release and containment approaches that allow careful evaluation in controlled situations might be explored, as governance issues are addressed.

### 2.6. Bacteria Could Potentially Be Engineered to Draw Down Atmospheric Carbon

In principle, it is possible to engineer nonphotosynthetic bacteria to utilize atmospheric CO_2_, rather than sugar, to create biomass. As a proof of principle for a model bacterium, an *Escherichia coli* strain that produces all its biomass from atmospheric CO_2_ using formate oxidation as an energy source has been engineered in the Milo Lab [34], thereby demonstrating that microbes can be used to help draw down atmospheric carbon. Among the remaining challenges is reducing the level of respired carbon below that of utilized carbon and economic scaling of treatment systems that do not impact natural ecosystems. In addition to engineering model bacteria such as *E. coli* to utilize CO_2_, the opportunity exists to engineer native C1 utilizers (e.g., acetogens and Cupriavidus) to be more efficient or develop completely synthetic CO_2_ fixation pathways/organisms, as demonstrated by [35].

### 2.7. Bacteria Could Potentially Be Engineered to Decrease Atmospheric Methane

Although the concentration of methane in the atmosphere is about 2 orders of magnitude lower than that of CO_2_, the impact of a pulse emission radiative force—the extent to which it increases the difference between inward and outward energy flux to and away from the planet—is about 25-fold greater than that of CO_2_ when averaged over 100 years and about 80-fold greater than CO_2_ when integrated over its atmospheric lifetime of one to two decades. Consequently, diminishing methane emission from its various sources must be an important component of climate control [36] provided that rapid reduction in CO_2_ emissions is simultaneously accomplished [37].

Just as *E. coli* and other microbes can be engineered to utilize CO_2_ as a source of carbon for producing biomass, so too can methanotrophs—methane-oxidizing microbes—convert methane to methanol as their first biochemical step. During the last 10-15 years, new easy-to-culture methanotroph strains have been developed, for which almost all the tools used for *E. coli* can be exploited. Also, the opportunity exists to engineer model bacteria such as *E. coli* to utilize methane. Progress is rapid enough so that the possibility of engineering methane-sequestering bacterial communities appears promising [38] and deployment of treatment systems at a removal scale of 10 TgC/yr is potentially feasible.

### 2.8. Microbes Could Potentially Be Engineered to Produce Useful Products

Emerging metabolic engineering methods can be used to increase the capacity of microbes that would function as versatile factories for converting specified inputs into specified and useful outputs [39]. This possibility includes engineering enzymatic reaction pathways to produce novel and useful compounds such as fuels and other chemical commodity products (e.g., precursors of polymers and plastics) or manipulating host metabolism, as a way of optimizing flux distributions, to generate dedicated crops that have a low demand for fertilizer, water, and other useful products [15]. In principle, organisms might also be created that grow on plastic as a carbon source, thereby contributing to waste handling [40]. An example is the fermentation of carbon oxide-containing industrial waste gases (e.g., emissions from processing plants, steel industry, or refineries), which is already practiced at commercial scale (e.g., LanzaTech), though early in development.

Recent research suggests the possibility of carrying design even further, to the community level [41]. Such research explicitly recognizes the fact that microbes function as part of a complex ecology and draws upon the development of new mathematical methods and growing databases [42]. Although many challenges must be met and critical concerns addressed, it is possible that in the foreseeable future, it could become possible to program communication between and among cell populations to perform specified, novel functions in contained environments.

## 3. An Emerging Roadmap

Any roadmap must include plans for confronting and resolving serious ecological and societal challenges before potential technological solutions can be fully realized and deployed. It is easy to envision the development of technologies for drawing down atmospheric carbon, for bioremediation, or for increased crop yield, which could be effective—but they would also be of little help if they carry poorly understood risks. Perturbations of the carbon cycle on a global scale will be profound and irreversible in their consequences. Moving on a national agenda without serious and open analysis of risks and how to mitigate them would be a mistake both politically and ethically.

We need a much deeper understanding of managed ecosystem dynamics and how it impacts and is impacted upon by climate if we are to evaluate the potential effects of any mitigation strategy. One example is the coupling of nitrogen and phosphorous cycles in land ecosystems. An inadequate allowance for the linkage could lead to failure when trying to introduce engineered organisms with N-fixing capability into natural environments. Similarly, the impact of rapid CO_2_ drawdown on the symbiotic association between the multiple types of mycorrhizae fungi and the plant roots with which they associate and which play a critical role in carbon assimilation involves a complex dynamic that will require extensive experimental and computational research to achieve a reasonably predictive understanding.

(i) Decisions concerning the development and implementation of technologies that biologically manipulate ecosystems should be made on the basis of a careful assessment and evaluation of the benefits and risks of different options. This will require time and extensive experimentation as it is extremely difficult to model even small ecosystems accurately enough to understand perturbations
(ii) We need a much better understanding of biological engineering at very large scales involving communities of organisms. What can be engineered to work in a microbe in a Petrie dish may not work if scaled up to a bioreactor or an industrial fermenter. Some progress has been made in identifying and manipulating small communities of organisms that enable a particular environmental target to be achieved, such as nitrate reduction, carbon sequestration, or heavy metal contamination [43]. The experiences and expertise of the industrial and biopharmaceutical communities will be valuable and necessary here if SSB-engineered approaches are to be used. Unlike sugar fermentations that are typically carried out in batches, fermentation of carbon oxides or methane is typically carried out in a continuous fashion, yet outside the biopharmaceutical industry, the impacts of continuous fermentations for manufacturing are not well understood and modelled
(iii) The development of improved methods for containing replicating engineered plants and organisms must continue to receive high priority
(iv) The cost-benefit ratio, which varies with the type of technology, needs to be carefully analyzed since it will be central to any policy decisions
(v) It is important to note that the US and some other countries (e.g., China and some African countries) are more accepting/tolerant of genetically modified organisms (GMOs) than Europe. This underscores how important it will be to get the Ethical, Legal, and Social Implications (ELSI) and other societal acceptance efforts right. Climate change is a global challenge, requiring international efforts to determine how to work together effectively and harmonize different approaches. The scale of what is contemplated in terms of negative emissions by manipulating cultivars and forests is huge. For this mission to be successful, the Convention of Biological Diversity will have to be respected as well as its Cartagena and Nagoya Protocols. The ELSI-related R&D can proceed in parallel with the mitigation R&D so as to neutralize potential negative parts of the mitigation strategy and confirm the safety of the rest

There are many other scientific challenges and opportunities that were mentioned and will be fully discussed at the next international workshop. These include the following.

(i) The possible development and application of SSB for improved photosynthetic efficiencies and increased atmospheric CO_2_ capture
(ii) The consequences for an organism or an ecosystem, if an organism had a “better” form of photosynthesis
(iii) The possibility and desirability of safely introducing photosynthesis into currently nonphotosynthetic organisms
(iv) The possible alteration or adjustment of biomass distribution in multicellular plants, potentially increasing productivity
(v) The possible use of SSB technologies to redirect greater amounts of carbon into subsurface partitions
(vi) The possibility of engineering organisms in addition to *E. coli* to use CO_2_ as a primary energy source
(vii) The possible use of SSB technologies to increase methanotrophy
(viii) The development of biomes with controlled conditions that will enable assessment of SSB intervention strategies

In addition, a number of very important legal-societal questions need to be addressed, such as the following.

(i) How can we best prepare for unintended consequences of an intervention
(ii) Who is liable if an application of SSB technologies harms someone or some community
(iii) Will the hoped-for benefits be fairly distributed? Who will “win” and who will “lose”

As the scientific community begins to achieve reasonable confidence in the science, it must build a working relationship with the public. That means, among other things, public participation in meetings that might establish high-level planning agendas, better communication channels with traditional media, and better use of electronic media and methods to establish respect for diverse views and mutual understanding [44]. Multiple organizations are properly concerned about issues such as safety, containment, equity, irreversibility, and unanticipated consequences, among others. These must be considered and are not likely to be addressed quickly. As Nobelist Richard J. Roberts points out [20], even GMOs, which are well understood and can benefit hundreds of millions of malnourished people, are encountering strong resistance in some nations. The advantages of SSB-based GMO technologies, which enable the optimization of biosystems for simultaneous carbon sequestration and bioproduct production, along with enhanced biosafety through new technological innovations such as DNA-free gene editing [45] and transgene biocontainment [46, 47], can help with the public acceptance of GMO for climate change mitigation. Sometimes, new technology itself can help address the issues. For example, in the case of the GMO controversy, using DNA-free genome editing does not introduce foreign DNA [45].

The potential impact of genetic engineering aimed at improving the environment will be more complicated, and it is not nearly as well understood as the impact of GMOs. Such applications are much riskier and need to be acknowledged as such. Their implementation, even if targeted toward modifying a single organism such as a fern, will undoubtedly require a diverse science-public governance structure. In addition, we need to keep in mind and, as objectively and accurately as we can, assess the risks of novel actions against the understood risks of not acting.

The International Union of Conservation of Nature is an example of an organization that has taken an important step toward open and trustworthy governance. It has conducted a comprehensive analysis of the risks and opportunities of gene drives and synthetic biology [48] and recognizes the need for communication and understanding of the goals between communities that until now have been strangers to one another [49]. Common ground may be found through acknowledging risks, open communication, and explicit attention to deliberative democracy and governance that embraces all stakeholders: scientists, policymakers, and the public [50]. Engagement is a two-way street that involves the sharing of information, values, and opinions within a framework of mutual respect and shared decision-making [51].

Developing global decision frameworks around SSB will be complex and sometimes controversial because they must be made in the face of significant scientific uncertainty. An additional concern is that SSB could create unequally distributed risks/benefits. That is why it is imperative that researches examining the ecological and societal implications are sufficiently funded alongside any investment in the technologies themselves. From the outset, though, it must be clear to all, scientists and public alike, that implementation beyond the lab setting can happen only with robust international governance systems in place.

Finally, we close by noting the often overlooked obvious. The main challenge in fighting climate change can only be met by a combination of reduced GHG emissions and carbon drawdown strategies; any deployment of SSB or any other drawdown strategy cannot be seen as a substitute for required reductions. Indeed, SSB can also contribute to approaches to achieve overall sustainability. Ultimately, however, the challenge is neither technological, regulatory, nor ethical—it is developing and acting on a well-organized and effective plan. All the possibilities mentioned above, as well as many others, are being developed and supported by federal and private funds. The efforts, however, are not adequately organized, a process for achieving goals has not been well articulated, communication is fragmented, and financial support is far too meager. The 20th century witnessed extraordinarily challenging but eminently successful large-scale engineering projects either to meet a crisis (the Manhattan Project) or to stimulate major transformations in human knowledge (the Apollo Missions and the extremely contentious Human Genome Project). Addressing the world’s climate would do both.

We believe that a collaborative engineering project—with scale much larger than the Manhattan Project and Human Genome Project, involving scientists, policy experts, and ethicists from many countries across the world—will be required to bring SSB to bear upon the climate problem. This workshop was an important first step for priming the discussion on such an important subject, and plans are already underway for international follow-ups.

## Additional Points

*One Sentence Summary*. We report the deliberations of an interdisciplinary group of scientists, policy experts, and ethicists who met to evaluate the benefits and risks of employing synthetic and systems biology as part of a climate change mitigation strategy.
